# Revolutionizing healthcare by use of artificial intelligence in esophageal carcinoma – a narrative review

**DOI:** 10.1097/MS9.0000000000001175

**Published:** 2023-08-15

**Authors:** Anmol Mohan, Zoha Asghar, Rabia Abid, Rasish Subedi, Karishma Kumari, Sushil Kumar, Koushik Majumder, Aqsa I. Bhurgri, Usha Tejwaney, Sarwan Kumar

**Affiliations:** a Karachi Medical and Dental College; b Ziauddin University; c Liaquat College of Medicine and Dentistry; d Dow University of Health Sciences; e Jinnah Sindh Medical University, Karachi; f Shaheed Muhtarma Benazir Bhutto Medical University, Larkana, Pakistan; g Chittagong Medical College; h Department of Medicine, Chittagong Medical College, Chittagong, Bangladesh; i Valley Health System, New Jersey; j Wayne State University, Michigan, USA; k Universal College of Medical Sciences, Siddharthanagar, Nepal

**Keywords:** (MeSH terms): esophageal neoplasms, AI (Artificial Intelligence), cancer of esophagus

## Abstract

Esophageal cancer is a major cause of cancer-related mortality worldwide, with significant regional disparities. Early detection of precursor lesions is essential to improve patient outcomes. Artificial intelligence (AI) techniques, including deep learning and machine learning, have proved to be of assistance to both gastroenterologists and pathologists in the diagnosis and characterization of upper gastrointestinal malignancies by correlating with the histopathology. The primary diagnostic method in gastroenterology is white light endoscopic evaluation, but conventional endoscopy is partially inefficient in detecting esophageal cancer. However, other endoscopic modalities, such as narrow-band imaging, endocytoscopy, and endomicroscopy, have shown improved visualization of mucosal structures and vasculature, which provides a set of baseline data to develop efficient AI-assisted predictive models for quick interpretation. The main challenges in managing esophageal cancer are identifying high-risk patients and the disease’s poor prognosis. Thus, AI techniques can play a vital role in improving the early detection and diagnosis of precursor lesions, assisting gastroenterologists in performing targeted biopsies and real-time decisions of endoscopic mucosal resection or endoscopic submucosal dissection. Combining AI techniques and endoscopic modalities can enhance the diagnosis and management of esophageal cancer, improving patient outcomes and reducing cancer-related mortality rates. The aim of this review is to grasp a better understanding of the application of AI in the diagnosis, treatment, and prognosis of esophageal cancer and how computer-aided diagnosis and computer-aided detection can act as vital tools for clinicians in the long run.

## Introduction

Highlights

- The paper discusses the rising incidence rates of esophageal cancer globally and the regional disparities, emphasizing the importance of ethnicity, genetic factors, and lifestyle in its development.
- The role of artificial intelligence (AI) techniques, specifically deep learning (DL) and machine learning, in improving the identification and characterization of precursor lesions is explored. DL helps in computer-aided detection and computer-aided diagnosis, while machine learning can identify cancerous or benign lesions directly.
- The challenges in the current state of diagnosing esophageal cancer are highlighted, including the inefficiency of conventional endoscopy, the difficulty in identifying high-risk individuals, and the poor prognosis of the disease. AI can help in detecting endoscopically invisible lesions and reducing missed cancer rates caused by human factors.
- The application of AI in the diagnosis of esophageal cancer is discussed, with various studies showcasing promising results in using DL and CNNs to classify and segment esophageal lesions. The diagnostic accuracy of AI has been found to be high, potentially reducing miss rates and enabling early-stage cancer diagnosis.
- The paper also explores the role of AI in the staging, prognosis, and treatment of esophageal carcinoma. The TNM staging system, commonly used for assessing disease severity and estimating prognosis, can be enhanced with the use of artificial neural networks to predict outcomes. AI has the potential to improve treatment planning and patient outcomes.

Esophageal cancer is still a leading cause of cancer-related mortality, with incidence rates increasing more than sixfold globally^1^. It is the eighth most common cause of cancer-related deaths globally, associated with a poor prognosis^2^. With a distant site metastasis, according to the American Cancer Society, esophageal cancer has a 5-year survival rate of 6%. East Asia, eastern and southern Africa, and southern Europe have high rates of esophageal squamous cell carcinoma (ESCC)^3^. However, ESCC is uncommon in North America and other parts of Europe^4^. These regional disparities demonstrate the importance of ethnicity, genetic factors, and lifestyle in the development of ESCC. Barrett’s esophagus, a metaplastic change of the normal squamous mucosa of the esophagus to a columnar lining, is the sole known precursor for esophageal adenocarcinoma increases the chance of developing esophageal adenocarcinoma by 30- to 40-fold^5,6^. Another type of esophageal cancer comprising squamous cell carcinoma is preceded by low-grade and high-grade squamous cell dysplasia, managed by esophagectomy and lymph node dissection^7^. It is imperative to make significant efforts to improve the early detection of precursor lesions. Artificial intelligence (AI) can play a vital role as a second reader in the endoscopic scenario, improving the identification and characterization of precursor lesions and allowing for targeted biopsies or real-time decisions of endoscopic mucosal resection or endoscopic submucosal dissection^2^. A multicenter study of 123 395 upper gastrointestinal endoscopies revealed a 6.4% esophageal cancer miss rate^8^. Computer-aided diagnosis (CADx) can help to counteract some of the missed cancer rates caused by human factors such as weariness and lack of concentration. However, there exists lack of training and guidance about the methods adopted in clinical practices to take assistance from AI models to reduce missed diagnoses and improve treatment plans. This review aims to highlight the role of AI and how it is incorporated to develop deep learning (DL) and machine learning (ML) models to inform clinical practices.

## Methods

An extensive literature review was conducted by using relevant articles published in English language and all types of study designs were included to expand the scope of this narrative review. Search terms used on Pubmed/MEDLINE included ʻArtificial intelligence AND Barrett’s esophagus AND Computer assisted diagnosis OR Convolutional neural network OR Deep learning AND Esophageal cancerʼ. References of the articles were screened by title and abstracts to identify any relevant information that can support our topic. Further full text screening was done for previously published review articles to identify gaps in the literature and elaborate on the importance of this topic comprehensively.

## AI techniques and their role in gastrointestinal imaging

DL is an important technique used mainly in real-time AI diagnosis and characterization of upper gastrointestinal imaging (GI) neoplasia^9^. It is a subset of ML that does not require structured data to develop an algorithm and can automatically learn representations from raw data, such as images, without the need for manual feature engineering. DL can easily explore all the pixels beyond the reach of the human eye for all consecutive images in an upper GI endoscopy and provide the location of the lesion in a bounding box. DL uses convoluted neural networks (CNN), the fundamental architecture of which is made up of several layers that are in charge of feature extraction, mapping, data reduction, and classifying the input picture to the output classes. A CNN is composed of three essential components. In order to create a map of all the characteristics, the convolution layer must first extract the features from the pictures. The feature map’s dimensionality is then decreased via a pooling layer, enabling the network to identify varying input pictures from the same classes. Last but not least, the input picture is classified to a variety of output variables using a fully linked layer. The output for DL requires an extensive and a convoluted algorithm of pattern recognition. In theory, any decision-making process in the clinical setting should be driven by combinations of appropriate data features in the context of GI endoscopy^10^. The two main tasks required from DL are computer-aided detection (CADe) and CADx. CADe plays a role in the detection and localization of the suspected malignant lesion, and on the other hand, CADx can differentiate between two or more diagnoses. CADe has been used to detect Barrett’s esophagus, early squamous cell dysplasia, and early gastric cancer. CADx has been used to predict the depth of invasion of the tumor, presence or absence of malignant/precancerous lesions such as gastric atrophy, and differentiate between neoplastic and non-neoplastic lesions. A gastroenterologist can use a combination of both during an endoscopy to perform targeted biopsies, usually in the shape of an activation map^5^. However, extensive training and validated classification systems are required to employ CADe and CADx in routine practices. On the contrary, ML requires the development of algorithms that use structured data and can function directly without human input^11^. To summarize, ML will identify brand new images directly as cancer or benign (CADe), and DL will provide multilayer analysis with the classification of the lesion in question (CADx)^12^. Both can be used for classification tasks; however, DL uses a much more complex neural network.

## The current state of the diagnosis of esophageal cancer and its challenges

In gastroenterology, the primary disease diagnostic and monitoring method is white light endoscopic (100 000–400 000 pixels) assessment of the gastrointestinal system^13,14^. Other endoscopic modalities to increase visualization of mucosal structures and vasculature include narrow-band imaging (electronic chromoendoscopy) and white light endoscopy. Narrow-band imaging (NBI) uses blue light wavelengths (415–540 nm) to see hemoglobin (peak absorption 415 nm) and improve micro vessel contrast^9^. Endocytoscopy and endomicroscopy are recent endoscopic methods that allow a higher magnification to see better mucosal structures and histology-level visualization of intestinal disease^15,16^. However, conventional methods are partially inefficient in detecting esophageal cancer because not everyone with a cancer diagnosis has a precursor lesion. Studies have shown that dysplasia within BE lesions increases cancer risk significantly: the yearly risk is about 1% for people with low-grade dysplasia and more than 5% for those with high-grade dysplasia. However, 80–90% of esophageal adenocarcinoma cases are diagnosed in patients with no history of BE. Endoscopic screening detects BE in 6–12% of individuals with chronic GERD symptoms, most Caucasian men over 50^17^. According to Spechler and Souza^18^, patients with chronic GERD symptoms and at least one risk factor of esophageal cancer are candidates for a screening endoscopy, but 40% of patients with esophageal adenocarcinomas do not report a history of GERD. The main challenge in managing esophageal cancer is due to difficulty identifying people at high-risk and the disease’s poor prognosis. While cancers discovered through a BE surveillance program or as an incidental finding during a gastroscopy for another reason may be at an early-stage, the majority of esophageal cancers are discovered after symptoms such as dysphagia develop, and malignancies are locally advanced. As a result, only one out of every eight esophageal tumors are detected at an early-stage (T1)^19^. Typical symptoms occur only after 50% of the lumen size has been reduced which clinically presents as progressive dysphagia, weight loss (typically ≥ 10 kgs in 1 month), vomiting, and hematemesis (hematochezia and melena)^20^. High-resolution white-light endoscopy reveals mucosal abnormalities during gastroscopy. If erosions, ulcers, strictures, or metaplasia are seen, the endoscopist must determine if the cause is non-neoplastic or neoplastic. Discolorations, thin granular surfaces (orange peel look), and little elevations and troughs in the Barrett layer are all dysplastic indications. High-grade dysplasia is characterized by a landscape form and distinct erosions^21^. The drawback of this technique is a misdiagnosis of the lesions, which can be overcome by DL algorithms that can differentiate between various dimensions and layers of a small lesion and ML that can accurately diagnose if it is neoplastic. Behrens *et al*. conducted a prospective cohort examining the context and technique of detecting early neoplasia in BE during routine outpatient endoscopy. Three primary findings were found: (i) In patients with short-segment Barrett’s esophagus, almost all early tumors are diagnosed using index endoscopy rather than Barrett’s surveillance; (ii) approximately 40% of all early neoplasia are endoscopically invisible and can only be diagnosed using four-quadrant biopsies; and (iii) the macroscopic tumor type has a significant influence on the detection rate for neoplasia^22^.

This is where AI can be implicated in identifying endoscopically invisible lesions. Despite the diagnostic accuracy of upper GI endoscopy, it is not uncommon to miss a neoplastic lesion due to various reasons highlighted by Menon *et al*., which reported that up to 11.3% of upper GI malignancies are missed at endoscopy up to 3 years before diagnosis. Some reasons are male sex, presentation with alarm symptoms, endoscopists with less experience, pathology errors, failure to adequately biopsy lesions, follow-up errors, and ESCC are all important factors in the failure to detect cancer at upper gastrointestinal endoscopy^23^. Luo *et al*. conducted an extensive meta-analysis to study the accuracy of AI-assisted diagnosis of upper GI malignancy in gastric cancer. All findings included AI exhibiting an excellent diagnosis, high accuracy, sensitivity, and specificity^24^. Diagnosis of a GI malignancy requires years of expertise, skill, and expert biopsy techniques with exceptional clinical judgment, which many gastroenterologists lack, especially in low-resource settings. As well as high quality or high-resolution endoscopy machines and a manual diagnosis can often be misleading for missing a premalignant lesion. A multimodality approach is taken to treat esophageal cancer, which includes neoadjuvant chemoradiotherapy or perioperative chemotherapy in addition to esophageal resection and is becoming more popular worldwide due to a survival benefit over surgery^25–28^. Although esophagectomy remains the foundation of curative treatment for early-stage esophageal cancer, it is still associated with significant postoperative morbidity, despite promising results from minimally invasive techniques. In this context, physical condition and reaction to neoadjuvant treatment may be crucial determinants in determining which patients may benefit from surgery. Furthermore, the entire perioperative trajectory must be optimized from the initial outpatient clinic visit to postoperative discharge^29^. Early detection of neoplasia will also allow organ preservation and reduce the morbidity associated with surgical management.

## Artificial intelligence and its application in the diagnosis of esophageal cancer

Recent years have seen great advancements in AI, particularly in DL, which has achieved unparalleled success in a variety of disciplines with revolutionary effectiveness on par with human capabilities^30^. Recently, there has been a trend toward using DL in healthcare and exploring its clinical implications^31,32^. As a subfield of AI, DL employs many layers of neurons to identify abstract patterns from the input. DL offers promising potential in image analysis tasks such as segmentation, categorization, and prognosis^33–35^. Much literature has created DL approaches for medical image analysis, such as ultrasound, CT, MRI, and X-ray^36–38^. In recent times, DL has been gradually used in endoscopic image analysis of the colon, stomach, and intestine, among other places, with promising results in identifying and diagnosing diseases like tumors, polyps, and ulcers^39–41^. Several studies have applied DL to classify and segment esophageal lesions^42–45^.

Mendel *et al*.^46^ used a migration-based learning method to segment endoscopic pictures which contained cancer and BE. Endoscopic pictures of malignancy, BE, and inflammation was segmented using a CNN by Wu *et al*.^42^. Pan *et al*.^47^ developed a fully automated method for segmentation and identification of Barrett’s esophagus in endoscopic images by including 443 images from 187 individuals as well as DL methods to identify gastroesophageal junctions and squamocolumnar junction, respectively. Wu *et al*. developed an esophageal lesion network for individual esophageal lesions’ classification and segmentation using deep CNN, which achieved classification with a sensitivity of 0.9034, specificity of 0.9718, and accuracy of 0.9628, and the segmentation with a sensitivity of 0.8018, specificity of 0.9655, and accuracy of 0.9462. These indicate factors indicated that this model was an efficient, accurate, and reliable tool for diagnosing esophageal lesions in clinics^48^. With encouraging results, several computer-aided diagnoses (CAD) systems have recently been tested in upper GI endoscopy. AI has the potential to successfully aid both trainees and experienced physicians in reducing variability in the identification of esophageal cancer, therefore enhancing diagnostic accuracy independent of individual competence and essentially eliminating the existing constraints of EGDS^49^. Arribas *et al*.^50^ did a literature review of 19 studies, including 218 patients of esophageal squamous cell neoplasia, 445 of Barrett’s esophagus-related neoplasia, and 433 patients of gastric adenocarcinoma which yielded an overall sensitivity of 90% and specificity of 89%. Overall, a high diagnostic accuracy for AI was found, which can substantially reduce miss rates and diagnose cancer in its early stages. Similarly, over the years, larger data sets have been produced, supporting the overall diagnostic accuracy of AI and producing promising results. In 2016, Liu *et al*.^51^ developed a combined diagonalization principal component analysis method that successfully recognized 90.75% of esophageal cancer with an area under the curve of 0.9471. It was Horie *et al*. who made the first attempt to apply DL to diagnose ESCC using a large number of endoscopic images to improve the performance of the CAD system. The CNN exhibited 99% diagnosis accuracy for ESCC, 92% for superficial cancer, and 92% for advanced cancer. The sensitivity of CNN was 97% at or prepatient level and 77% at the preimage level^52^. Cai *et al*.^53^ adopted a mixed DNN-CAD model that detected 91.4% of early precancerous lesions, higher than experienced endoscopists. Ohmori *et al*. used a CNN based on a single shot multi-box detector to recognize SCC in both magnified endoscopy (ME) and non-ME pictures [including white light imaging (WLI) and NBI/blue laser imaging (BLI)]. ME, non-ME + WLI, and non-ME + NBI/BLI accuracy was 77, 81, and 77%, respectively, with high SEN and moderate SPE. The outcome was comparable to those of experienced endoscopists examined in this study which suggests the adaptability of this model, which can be used to establish a confident diagnosis^54^. Given the promising results and improved accuracy of AI-assisted diagnostic techniques, physicians, and healthcare setups across the globe should adopt its use further in their clinical practices.

## Artificial intelligence and its role in the staging, prognosis, and treatment of esophageal carcinoma

The International Union Against Cancer TNM categorization system is based on the anatomic carcinoma invasion and metastasis degree. The TNM categorization system aims to offer doctors a platform for assessing the amount of disease, assisting in treatment planning, and estimating prognosis. It also requires interdisciplinary efforts where gastroenterologists, radiologists, oncologists (medical and surgical), and histopathologists play an equal role. The TNM staging method has long been used to determine patient outcomes in esophageal carcinoma and other cancers^55^. Fumiaki *et al*. applied artificial neural networks (ANN) to identify colorectal and esophageal carcinoma progression. The ability of ANN and Linear Discriminate analysis (LDA) models to predict 1-year and 5-year outcomes was evaluated using just TNM staging information. For 1-year survival and 5-year survival, the area under receiver operating curve values produced from ANNs were 0.7697 [standard error (SE)=0.0251] and 0.8061 (SE=0.0279), respectively. In contrast, the area under receiver operating curve values derived from LDA were 0.7406 (SE=0.0272) and 0.7626 (SE=0.0313), respectively. Thus, when using TNM staging criteria, ANNs outperformed LDA in predictive accuracy^56^. This study was a cornerstone in establishing ANNs as powerful predictive tools for esophageal cancer. Knabe *et al*. used 1020 images from 77 patients with Barrett’s adenocarcinoma for the training and convolution of a deep neural network. Out of 1020, 821 images were selected to train their model. A model to accurately T-stage Barrett’s adenocarcinoma was developed, further establishing AI as an effective staging tool. In their study, AI correctly identified Barrett’s mucosa without neoplasia with an accuracy of 85%. Mucosal cancer was detected with 72% (95% CI: 67.5–76.4), 64% (95% CI: 60.0–68.4), and 68% (95% CI: 64.6–70.7) sensitivity, specificity, and accuracy. For early Barrett’s neoplasia, the sensitivity, specificity, and accuracy were 57% (95% CI: 51.8–61.0), 77% (95% CI: 72.3–80.2), and 67% (95% CI: 63.4–69.5), respectively. With a sensitivity of 71% (95% CI: 65.1–76.7) and a specificity of 73% (95% CI: 69.7–76.5), more advanced phases (T3/T4) were detected correctly. The total accuracy was 73%^57^. George Sgourakis *et al*. performed an analysis pooling the effects of outcomes of 2098 patients enrolled in 37 cohort studies that used neural networks as data abstraction techniques. This method accurately identified the staging and metastatic spread to lymph nodes of esophageal cancer^58^. Hence, this suggests that ANN can serve as a potential tool for TN staging and lymph node involvement, which will determine the extent of surgical resection of the tumor. In another study, Zhang *et al*. used an AI-based CAD model, the goal of which was to create an AI-based computer-aided diagnosis system (AI-CAD) that simulated radiologists’ diagnostic logic for lymph node metastasis in ESCC patients to which consequently contributed to clinical treatment decision-making. A total of 689 ESCC patients having PET/CT scans were enlisted from three institutions and split into two external validation cohorts and a training cohort. For pretraining the model, 452 CT images from three publicly available datasets were also used. This model achieved an accuracy of 0.744 for predicting lymph node metastasis which was in line with cohorts derived from human expertise. With the aid of AI-CAD, human diagnostics was significantly improved^59^. The evidence across current literature shows the ability of AI-CAD models, neural networks, and DL networks to dictate management and treatment by providing accurate staging and prognostic details, which are crucial for a clinician and guide multimodality treatment is the mainstay of the approach in patients with esophageal cancer. A comparative study by Tokai *et al*. further supported the effectiveness of applying convolutional neural networks in determining the depth of invasion in ESCC. In 10 s, the AI-diagnostic system recognized 95.5% (279/291) of the ESCC in test photos, analyzed the 279 images, and correctly calculated the ESCC invasion depth with a sensitivity of 84.1% and an accuracy of 80.9%. This system’s accuracy score topped that of 12 of 13 board-certified endoscopists, and its area under the curve was larger than that of all endoscopists^60^.

When it comes to the treatment of any malignancy, many factors such as comorbids, progression of the disease, family history, adherence of the patient, the spread of neoplasia, local area involvement, and tolerance to chemotherapy, have a role to play and deciding treatment regimens for such patients varies from patient to patient. Perhaps, we can confer that these reasons explain the scarcity of data regarding AI and its application in treatment regimens for esophageal cancer. The development of the initial treatment strategy directly influences the care of esophageal cancer patients. A common difficulty is selecting which combination of systemic drugs, radiation treatment, and surgery is best for individuals with esophageal cancer at various stages. The ability to predict radiation sensitivity will aid in developing this approach. DL can evaluate multidimensional data streams in genomics and produce accurate radiation sensitivity predictions on data containing radiometric indicators. This discovery is the primary focus of current research on using AI in esophageal cancer. It can be a foundation to build on regarding its use in determining treatment modalities^60^. Table 1 summarizes major observational studies that used patient level data to develop predictive models in esophageal cancer and the results they yielded.

**Table 1:** Predictive models employed in prognosis and treatment of esophageal cancer.

References	Study design	Objective	Primary results
Luo *et al*.^61^	Multicenter case–control	Develop and validate GRAIDS – a system that analyzes clinical data from endoscopies	GRAIDS achieved high diagnostic accuracy with sensitivity similar to that of expert endoscopists and superior to that of nonexpert endoscopists. The negative predictive value was 0·978 for GRAIDS, 0·980 for the expert endoscopist, 0·951 for the competent endoscopist, and 0·904 for the trainee endoscopist.
Cui *et al*.^62^	Randomized control trial	Construct machine learning models for predicting survival of patients with ESCC by combining contrast enhancing CT images with clinical and radiological features	Combined models performed better with an accuracy of 70% in predicting the PFS and overall survival. It can be used as a decision marking-reference for clinicians.
Hu *et al*.^63^	Retrospective cohort study	To validate the performance of CT-based optimal model using DL for predicting response to neoadjuvant chemotherapy in ESCC	The optimal model achieved an AUC and accuracy of 0.805 and 77.1%, compared with 0.725 (0.605–0.846) and 67.1% (54.9–77.9%) for the radiomics model. All the radiological models showed better predictive performance than the clinical model.
Luo *et al*.^64^	Retrospective cohort study	Develop a nomogram model for predicting LPFS in ESCC patients treated with CCRT	The C-index of the nomogram was 0.745 in training cohort and 0.723 in validation cohort. The 3-year LPFS rate predicted by the nomogram model was highly consistent with the actual 3-year LPFS rate.
Peng *et al*.^65^	Retrospective cohort study	Explore the use of preoperative CT radiomics in predicting lymphovascular invasion (LVI) in esophageal squamous cell carcinoma (ESCC).	A total of 1218 radiomic features were extracted to construct a full-volume radiomics predictive model. According to LGOCV, the full-volume model showed the highest mean AUC for both the training and validation cohorts.
Cui *et al*.^66^	Retrospective cohort study	Investigate the predictive power of CT-based radiomics combined with genomics for the treatment efficacy of dCRT in ESCC patients.	Combined radiomics and genomics model predicted the best PFS for patients receiving dCRT using a Rad score and HRR pathway alterations.
Chu *et al*.^67^	Retrospective cohort study	Develop optimal model based on the 1 mm-isotropic-3D contrast-enhanced StarVIBE MRI sequence combined with clinical risk factors to predict survival in patients with ESCC.	A combined predictive model based on MR Rad-S and clinical risk factors had better predictive efficacy than the radiomics models alone for patients with ESCC. The optimal model showed highest performance in both training and validation groups for predicting DFS and OS.
Liu *et al*.^68^	Retrospective cohort study	Develop a combined predictive model for BES after SIB with CCRT in patients with ESCC	A nomogram derived from both radiomics signature and clinical prognostic factors showed favorable predictive accuracy for BES in ESCC patients who received SIB with chemotherapy.
Kong *et al*.^69^	Retrospective cohort study	Develop a predictive model using enhanced CT examination, and evaluate its clinical value for detecting LRFS in cases of ESCC after radiotherapy	A radiological label successfully predicted the LRFS of ESCC after radiotherapy. The radiomics nomogram complemented the clinical prognostic features and can improve the prediction of the LRFS after radiotherapy.
Takahashi *et al*.^70^	Retrospective cohort study	Develop and evaluate a radiomic model of [18F] FDG-PET/CT to PFS of dCRT for patients with esophageal cancer.	The [18F] FDG-PET/CT radiomic model can be used to predict PFS for patients with esophageal cancer who received dCRT.

AUC, area under receiver operator curve; BES, benign esophageal stricture; CCRT, concurrent chemotherapy; dCRT, definitive chemotherapy; ESCC, esophageal Squamous cell carcinoma; GRAIDS, gastrointestinal artificial intelligence system; HRR, homologous recombination repair; LGOCV, leave group out-cross validation; LPFS, local progress-free survival; LRS, locoregional recurrence-free survival; PFS, progression-free survival; ROI, regions of interest; SIB, simultaneous integrated boost.

## Ethical considerations in artificial intelligence

Privacy and surveillance, bias or discrimination, and perhaps the philosophical question of the role of human judgment are among the legal and ethical challenges that society faces as a result of AI^71^. There is no doubt that AI has the potential to transform healthcare by generating new and important insights from the massive amounts of digital data generated during healthcare delivery^72^. But on the one hand, the main dilemma stems from a lack of accountability regarding who will take responsibility in cases of error, security breach, and mishandling of large data sets. To fully realize AI’s promise in healthcare, the following aspects must be considered: (1) informed permission to utilize data, (2) safety and transparency, (3) algorithmic fairness and biases, and (4) data privacy^73^. AI in healthcare must adapt to a constantly changing environment with frequent disturbances while adhering to ethical guidelines to protect patients’ well-being^74^. However, an easy, key component of determining the security of any healthcare software is the ability to test the software and recognize how the software would fail. ML-Health Care Applicants, on the other hand, can be a ʻblack boxʼ problem, with workings that are not apparent to assessors, physicians, or patients. Researchers should clarify how such outputs, as well as predictions, might be included in the research. This data assists in determining the cost of the scientific trial and informs scientific research^75^.

In the coming future, we can see that AI will be increasingly employed in healthcare, necessitating moral accountability. Data bias must be avoided by employing proper algorithms that are based on unbiased real-time data. Diverse and inclusive programming groups, as well as regular audits of the algorithm, including its implementation in a system, are required. While AI cannot completely replace clinical judgment, it can assist clinicians in making better decisions. If there is a lack of medical expertise in a resource-constrained setting, AI might be used to undertake screening and evaluation.

## Challenges of the use of artificial intelligence in esophageal carcinoma

The main challenges in AI besides ethical dilemmas also include the limited understanding of deep neural networks and consistency in data extraction for developing DL models. Another possible challenge is the asymptomatic nature of early disease lesions, which causes patients to progress to late stages without a diagnosis and then the utility of AI can potentially decrease and healthcare workers would possibly not consider diagnostic accuracy as a priority, but rather a treatment and management will be preferred. Some drawbacks of applying AI, in the long run, can be that the endoscopists who train with AI-enabled technologies may grow reliant on AI for both diagnostic and therapeutic endoscopy. It is critical to avoid detraining endoscopists’ cognitive abilities. Although current research indicates that AI has great potential for assisting in endoscopy diagnosis, there are ʻblack boxesʼ in the logic of DL algorithm decision-making processes that humans find difficult to understand or comprehend. AI, like humans, may make mistakes. These may be erratic and unexplainable. It may be beneficial to employ AI for preliminary screening, finding regions of interest, and predicting histology, but the final choice should be made by people, attaining human–computer collaboration in practice. The existing AI technology can equal an endoscopist’s diagnostic skills and, with time, may be developed to outperform specialists in the area^76^. Besides, the cons of AI the pros of adapting AI-assisted diagnostic techniques overweigh the pitfalls and will be widely adopted in the future.

## Conclusion

Extensive literature reviews, pooled analyses, cohort studies, and correspondences focusing on the use of AI in guiding the treatment and diagnosis of esophageal cancer exist. However, there is a gap between its practical use, accessibility, and understanding of clinicians of AI-assisted diagnostic models. Furthermore, esophageal carcinoma is highly prevalent in China and Japan, these two countries, spear-headed various initiatives in adapting AI in the diagnosis and treatment of esophageal carcinomas. However, it is still of limited use in the remaining parts of the world, and Western countries that also share a significant burden of the disease are yet to adapt to CADe and CADx models for early detection of esophageal cancer. It is imperative for the scientific community across the globe to mobilize their research in the direction that incorporates ML and AI-CAD models to reduce the disease burden and significantly improve the diagnostic accuracy of the neoplastic lesions in esophageal cancer as with the advent of these tools it is foreseeable that the mortality and morbidity associated with this disease can be greatly reduced.

## Ethical approval

Ethics approval was not required for this review.

## Consent

Informed consent was not required for this review.

## Sources of funding

No funding recieved.

## Author contribution

A.M. and Z.A.: designed the study and conceived the idea; K.M., R.A., R.S., A.M., and Z.A.: wrote the first draft; K.K., S.K., and K.M.: wrote the second draft; A.I.B., U.T., and S.K.: worked on the revisions. All authors equally contributed to this work.

## Conflicts of interest disclosure

The authors declare that they have no conflicts of interest.

## Research registration unique identifying number (UIN)

It is not a human study.

## Guarantor

Koushik Majumder.

## Data availability statement

Not applicable.

## Provenance and peer review

Not commissioned, externally peer reviewed.
